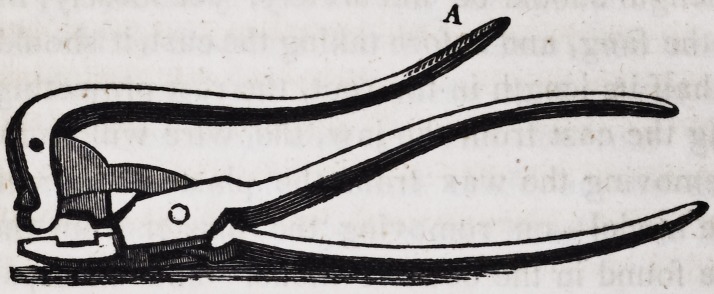# Contributions to Operative and Mechanical Dentistry

**Published:** 1844-03

**Authors:** W. H. Elliot

**Affiliations:** Fellow of the American Soc'y of Dental Surgeons.


### ARTICLE III.

*Contributions to Operative and Mechanical Dentistry.* By W. H. Elliot, Fellow of the American Soc'y of Dental Surgeons.

No. 3.

### ON THE PIVOTING METHOD OF INSERTING ARTIFICIAL TEETH.

This particular branch of the dental art is so well understood at the present day among the more enlightened members of the profession, that there are few errors in general practice to be corrected. I shall therefore confine myself more particularly to the instruments with which the operation is performed.

*Cutting Forceps.*—The excision of a tooth is an operation which cannot be safely performed with the instruments in common use, especially if the neck of the tooth be sound and healthy. Dr. Harris recommends filing the tooth about half off, before the forceps be applied; this, when practicable, renders the operation less liable to accidents, but when filing is impracticable, there remains but one alternative,—the operation must be performed with the forceps, be the consequences what they may.

It must be evident to every practitioner, that the profession should be supplied with an instrument which can be used on all occasions without risk of injuring the vitality of the root, by the violence of the concussion, or by wrenching the tooth from its natural position during the operation. To effect this, the instrument must be sufficiently strong to prevent it from springing; it must have a multiplied lever power, so as to require less effort on the part of the operator; and lastly, it must be so constructed that any irregularity in the motion of the hand, which gives the force, will have no effect on that part of the instrument which is applied to the tooth.

The instrument used by the writer, is a common cutting forceps, with the addition of a steel shaft, about three inches in length, and one-fourth inch in diameter, having upon one end a handle like that of a common key instrument, and upon the other end an elongated head, about one-half inch in length, standing like the handle at right angles with the shaft. One extremity of this head, is attached to one of the handles of the forceps about four inches from the joint of the instrument, by means of a little stirrup or link; the other extremity of the head is attached to the opposite handle by the same means. One or both of the stirrups should pass through the handles of the forceps, and be fastened by means of a screw-nut, so that the capacity of the instrument may be altered. In use, the instrument is placed upon the tooth, and held there by the left hand, while a rotary motion is given to the shaft with the right, which brings the handles of the forceps together with a force almost incalculable. The stirrups which connect the shaft with the handles of the forceps, should be made to play about loosely, so that any irregularity in the motion of the right hand, will not move the cutting part of the instrument.

*Destroying the Nerve*—May be most effectually done, by plunging into the cavity a bristle or bit of whale-bone, large enough to fill it. Either of these materials are preferable to a metallic instrument, for the reason that their elasticity will allow them to follow the canal of the nerve, although they may not have received the right direction from the hand. The amount of pain given depends entirely upon the length of time occupied in plunging and withdrawing the instrument.

*Filing the Root.*—Much pain and uneasiness may be saved the patient by holding the finger nail or some thin instrument against the end of the fang, as long as any of it remains above the gum; and afterward by pressing gently with the finger upon the gum opposite the root.

Aside from the condition of the root, a greater part of the irritation produced by the use of the file, depends upon the weight of that instrument, therefore the round or oval file should be abandoned for the thin file having one plane and one convex surface.

*Drilling the Root.*—Of all instruments ever used for this purpose, the common four or five sided broach is the least fit. Being as large or larger above than it is at the point, the slightest irregularity of the hand is keenly felt by the patient; and without a possibility of clearing itself, it forces its own chips in advance against the wounded nerve; thereby giving additional and unnecessary pain. Both of these evils may be remedied by making the drill of a proper shape.

The head or cutting part of the drill should be turned in the form of an egg, the small end serving for the point of the drill. This head should be filed into four thin teeth; the shank should be about one fourth as large in diameter as the head of the drill, so that it need not come in contact with the root while in use. When a greater number of teeth are made on the drill, it will not clear itself with facility, when a less number, it is liable to jar.

If a bow be used for propelling the drill, the handle should be placed in the centre, at right angles with it, so that a simple motion of the wrist will be sufficient, without moving the whole arm, as is the case with the common bow.

To prevent the slipping, or wearing of the cord, two cords should be used instead of one, so that one will be running on to the pully, while the other is running off, and vice versa, like the cylinder of a wooden clock. Each cord should also be provided with a guide.

*Fitting the Crown.*—A stone or cylinder about three inches in diameter should be used for this purpose, which will give the crown sufficient hollowness to prevent it from rocking on the root. To secure this object it has been recommended to sink the end of the fang around the orifice; this is certainly a very unscientific practice, for it not only forms a chamber for the retention of extraneous matter, but it places the natural decay of the root, a year or two in advance; for that portion of the stump which is removed by the counter sink, is the first to be attacked by disease.

*Materials for Pivots.*—In all cases where the root occupies a natural position, condensed hickory is the most suitable material for a pivot; unless indeed the root be broken or otherwise injured so that the crown cannot be brought to an accurate and firm bearing upon all parts of it. Under such circumstances the metallic pivot is the only one that will offer much resistance to lateral force.

In case a root occupies an unnatural position, I refer the reader to the very excellent method recommended by Dr. Solyman Brown.*

For the purpose of knowing exactly where the pivot should be soldered into the plate, a piece of iron wire three-eighths or half-an inch in length should be accurately, yet loosely, fitted to the opening in the fang, and before taking the cast, it should be placed with about half its length in the root, the rest projecting out. On withdrawing the cast from the jaw, the wire will be found in the wax—on removing the wax from the plaster, the wire will be found in the model—on removing the plaster from the lead, the wire will be found in the counter model—and on separating the two metals, the wire will be found in the tin model of the mouth, occupying its true position. The plate may now be prepared and slipped on to the wire which will hold it in its place while it is receiving the impression.

A metallic pivot may be fastened temporarily in the root by a bit of sheet lead, bent around it in the form of a tube, and of sufficient thickness to fill the orifice of the fang. For fastening the pivot permanently, the shavings of hickory, after being compressed between two smooth plates of iron, heated to about two hundred and fifty degrees Farenheit, may be used instead of lead.

In case it becomes necessary to give egress to matter through the root, the metallic pivot may be made in the form of a tube, or if hickory be used, a fine gold tube of sufficient calibre to receive a bristle, may be placed in the centre or side of the pivot, and an opening continuous with the tube should be made in the back of the artificial crown.

The practice of placing foil between the root and the crown is erroneous; for if it were possible to exclude the fluids of the mouth by such a course, the operation would most certainly fail. The pivot not being supplied with moisture, would not expand, and consequently would be liable to be displaced.

*Removing the Artificial Crown and Pivot.*—To remove the crown, a small instrument in the form of a double wedge may be

* Journal, vol. 2d, page 168.

forced in between it and the root, one half of the wedge passing upon one side of the pivot, and the other half upon the other side.

Should the pivot remain in the root instead of being brought away with the crown, it may be removed with an instrument like the cut below, without pulling upon the fang a single ounce.

When the jaws of the instrument are firmly fixed upon the pivot, it may be extracted by pressing down lever A.

In case the pivot be broken, and a part of it remaining in the root, a small pointed instrument resembling a twisted gimblet without a screw, may be used with safety. This instrument not only cuts rapidly, but follows the fibres of the wood, and is therefore easily directed, so as not to cut away the substance of the fang.

NOTE.—The writer begs leave to express his gratitude to Dr. Harris for the very gentlemanly manner in which he has treated his answer to the note appended to his first article. He is aware that the opinion of the annotator, when taken alone, is sustained by the authors quoted, yet when coupled as it is in the note, with the assertion, that he is in error in regard to the *originality* of his views, and used as it apparently is to prop that assertion, it may have a widely different meaning from that of the quotations in question, and therefore, in that case, cannot be supported by them.

If in complaining, he has complained too loudly, it is only attributable to pain he felt, at seeing his first offering upon the shrine of his profession, characterised by such an accusation.

### ARTICLE VI.

*Extracting Forceps and Key.* By W. W. H. THACKSTON, D. D. S.

## Figures and Tables

**Figure f1:**